# Postoperative Chemical Radiculitis Mimicking Recurrent Disc Herniation: A Case Report

**DOI:** 10.7759/cureus.106929

**Published:** 2026-04-12

**Authors:** Hamza Sriri, Mehdi Mdarhri, Oualid Hmamouche, Marouane Hammoud, Faycal Lakhdar, Mohammed Benzagmout, Chakour Khalid, Mohammed Chaouielfaiz

**Affiliations:** 1 Neurosurgery, Centre Hospitalier Universitaire Hassan II, Fez, MAR; 2 Neurological Surgery, Centre Hospitalier Universitaire Hassan II, Fez, MAR

**Keywords:** chemical radiculitis, lumbar spine surgery, magnetic resonance imaging, postoperative complication, radicular pain

## Abstract

Early recurrence of radicular pain after lumbar discectomy is commonly attributed to recurrent disc herniation; however, non-compressive inflammatory mechanisms such as chemical radiculitis should also be considered. We report the case of a 30-year-old patient who underwent L5-S1 discectomy for S1 radiculopathy with motor deficit, with an initially favorable postoperative course and complete pain relief. Three weeks later, the patient developed severe recurrent S1 radicular pain without any new neurological deficit. Magnetic resonance imaging suggested recurrent disc herniation, leading to surgical re-exploration, which revealed no evidence of recurrent herniation or hematoma but instead an inflamed and swollen S1 nerve root. The patient was subsequently managed conservatively, with progressive resolution of symptoms. This case highlights the importance of recognizing chemical radiculitis as a potential cause of early postoperative radicular pain to avoid unnecessary reoperation.

## Introduction

Lumbar discectomy remains the standard surgical treatment for symptomatic disc herniation, with favorable outcomes in most cases. However, early postoperative recurrence of radicular pain represents a diagnostic challenge requiring careful evaluation [1].

Although recurrent disc herniation is the most commonly suspected cause, other etiologies must be considered, including postoperative complications and inflammatory radicular conditions. Chemical radiculitis is likely underrecognized and may explain discordant clinico-radiological findings [2,3].

The nucleus pulposus becomes immunogenic when exposed to the epidural space and triggers an inflammatory cascade mediated by cytokines such as TNF-α and IL-1β, leading to nerve root irritation even in the absence of mechanical compression [4-6].

## Case presentation

**Figure 1:**
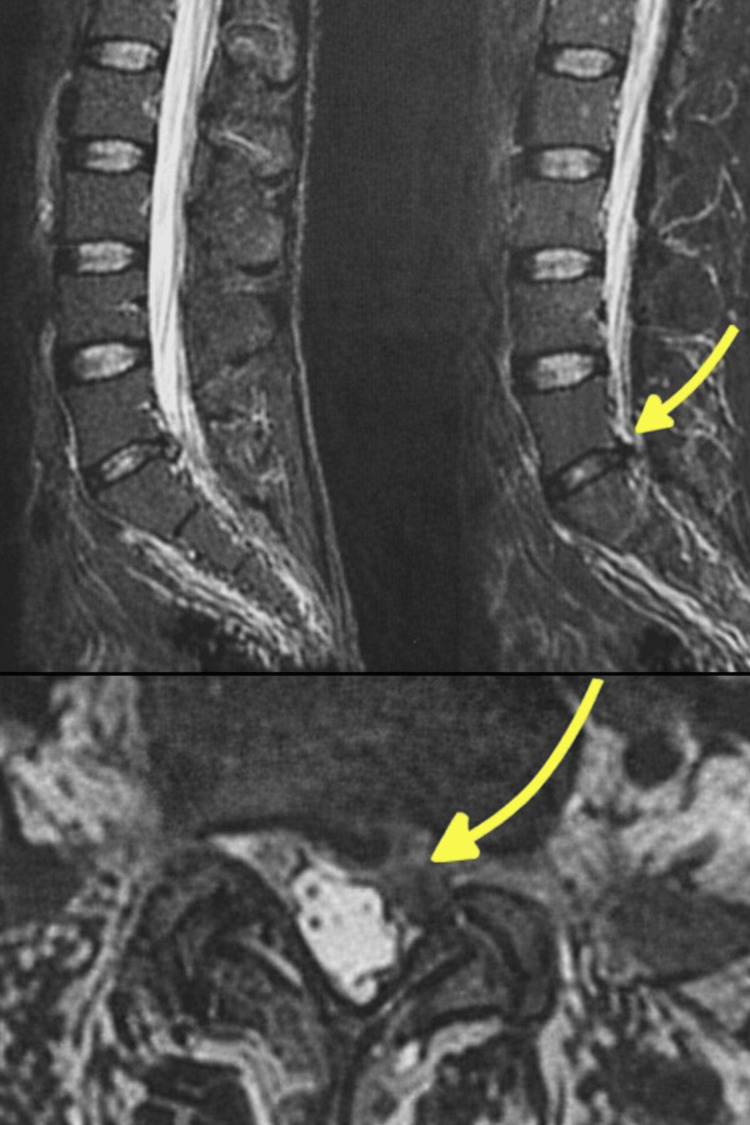
A sagittal and axial view of a lumbar MRI showing a large L5–S1 disc herniation.

**Figure 2:**
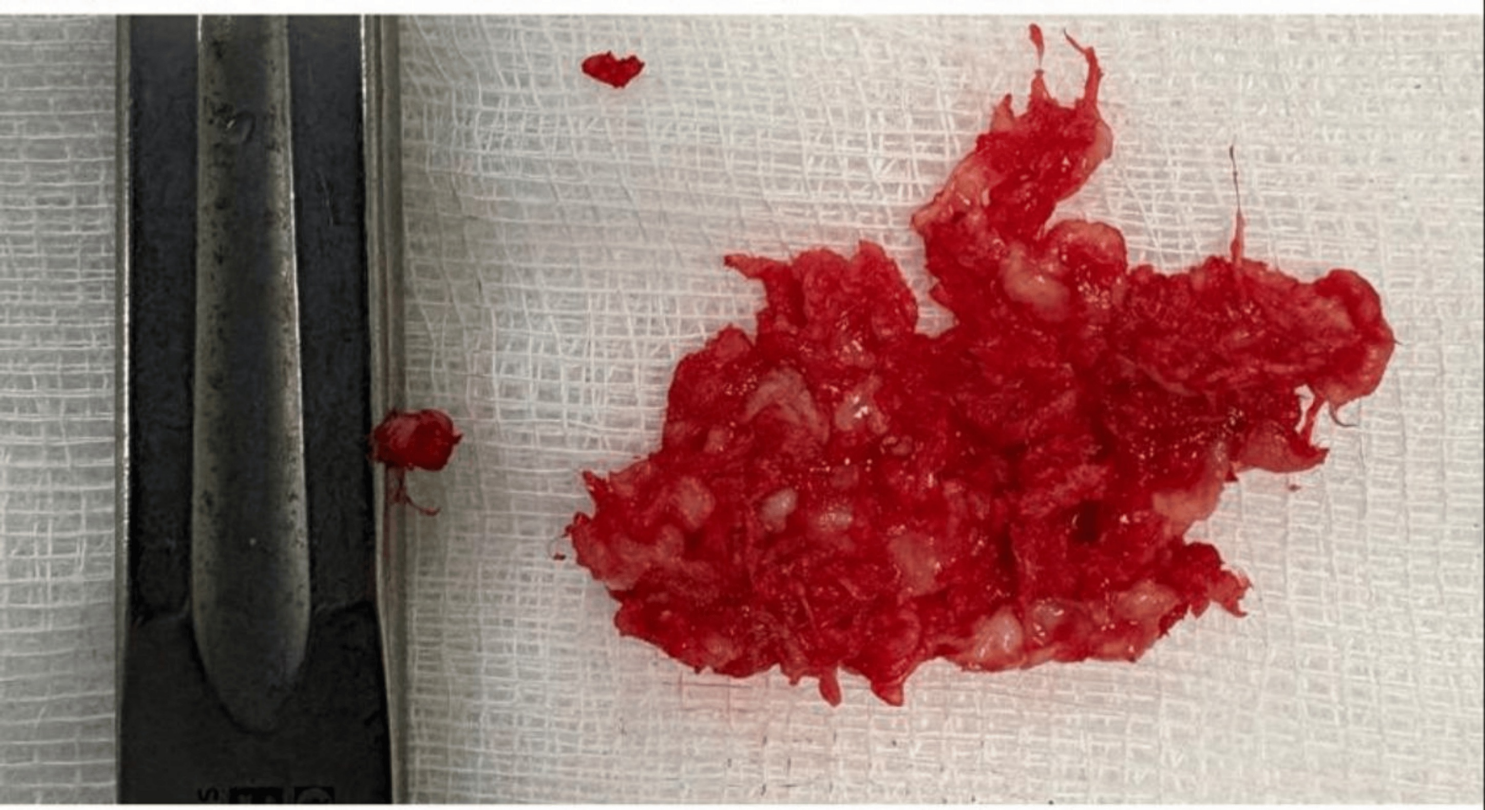
Intraoperative photograph of the excised L5–S1 disc fragment following lumbar discectomy. The specimen corresponds to degenerated nucleus pulposus material.

A 30-year-old male patient presented with S1 radiculopathy associated with a motor deficit graded 4/5 due to a large L5-S1 disc herniation causing significant compression of the S1 nerve root (Figure 1). The patient underwent lumbar discectomy with an initially favorable postoperative course, marked by complete resolution of pain and neurological improvement (Figure 2).

Three weeks after surgery, the patient developed severe recurrent S1 radicular pain. The pain was sharp, intense, and radiating along the S1 dermatome, exacerbated by standing and walking, without associated motor deficit or sphincter dysfunction.

Postoperative magnetic resonance imaging (MRI) showed equivocal postoperative changes at the surgical level with suspected recurrent left-sided L5-S1 disc material and persistent contact with the S1 nerve root.

**Figure 3:**
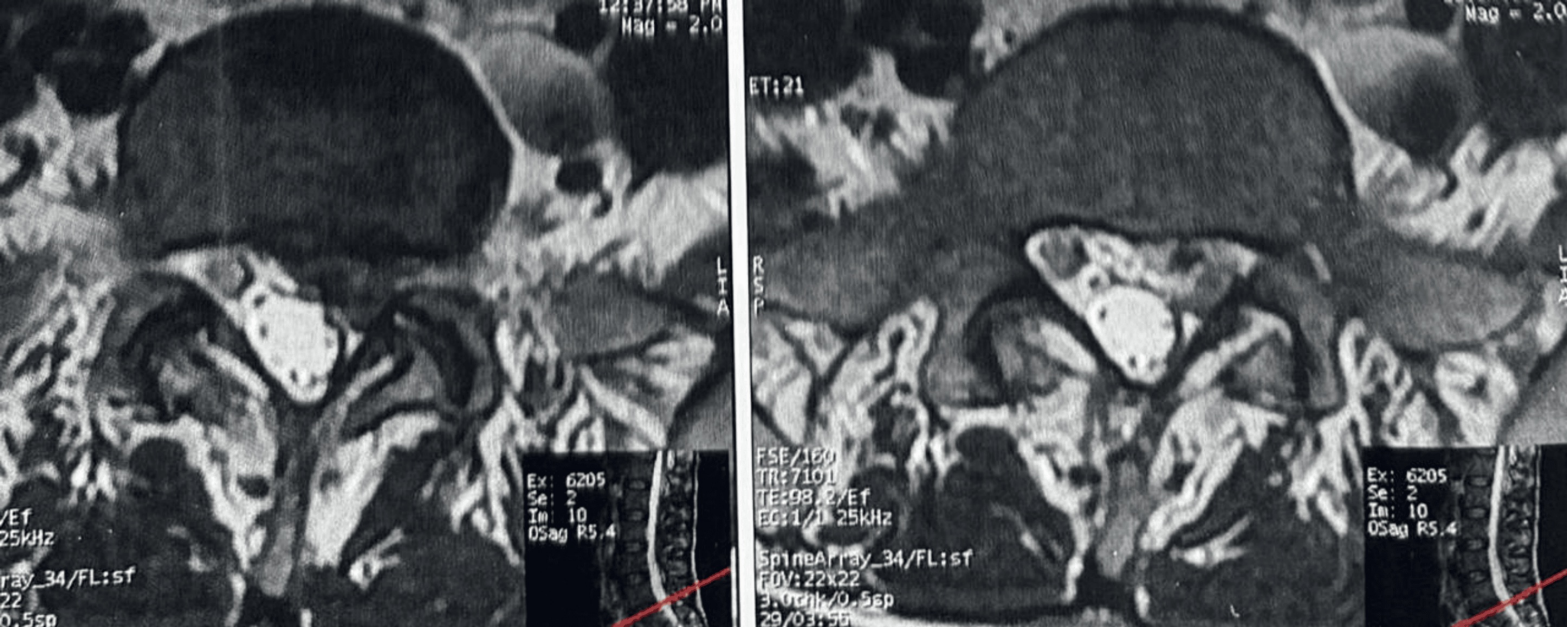
Postoperative axial lumbar MRI showing no evidence of recurrent disc herniation. The S1 nerve root appears enlarged and hyperintense, consistent with inflammatory radiculitis.

Given the severity of symptoms and diagnostic uncertainty, surgical re-exploration was performed. No recurrent disc herniation, epidural hematoma, or free fragment was identified. However, an inflamed and enlarged S1 nerve root was observed, consistent with chemical radiculitis (Figure 3).

Conservative treatment was initiated, including rest, corticosteroids, and neuropathic pain medication (gabapentin), followed by delayed physiotherapy. The patient showed significant clinical improvement.

## Discussion

Postoperative chemical radiculitis corresponds to an aseptic inflammatory reaction of the nerve root induced by exposure to nucleus pulposus material. Degenerated disc tissue releases pro-inflammatory mediators, including TNF-α, IL-1β, and prostaglandins, leading to nociceptive sensitization, nerve root edema, and altered neural conduction [3,5,6]. Experimental studies have demonstrated that direct contact between the nucleus pulposus and nerve roots is sufficient to induce radiculopathy even in the absence of mechanical compression [1,3].

Intraoperative factors, such as nerve root manipulation or prolonged retraction, may exacerbate this inflammatory response by increasing vascular permeability and neural susceptibility. Additional local mechanisms, including micro-edema or micro-hematoma, may further contribute to symptom severity [4].

Magnetic resonance imaging (MRI) is the modality of choice for postoperative evaluation; however, it has important diagnostic limitations in this context. Inflammatory radiculopathy may manifest as diffuse nerve root thickening and contrast enhancement, potentially mimicking recurrent disc herniation [7-9]. A substantial proportion of postoperative MRIs performed for suspected recurrence do not reveal compressive pathology but rather inflammatory or fibrotic changes [7-9]. In this setting, the differential diagnosis includes recurrent disc herniation, epidural hematoma, postoperative fibrosis, and chemical radiculitis, which may present with overlapping clinical and radiological features. Among these, epidural hematoma was considered unlikely in our case due to the absence of acute neurological deterioration, while imaging findings were not consistent with a well-defined recurrent disc fragment.

Clinically, recurrence of radicular pain after a pain-free interval is suggestive but not specific. Postoperative radiculitis appears to be relatively frequent and often resolves with conservative management, including corticosteroids and neuropathic pain medications [7-9]. Recognizing this entity is essential to avoid unnecessary surgical re-exploration.

From a clinical perspective, this case highlights the diagnostic challenge of distinguishing between recurrent disc herniation and chemical radiculitis in the early postoperative period. In our patient, the recurrence of severe radicular pain after an initial pain-free interval, associated with inconclusive MRI findings, led to a strong suspicion of recurrent disc herniation. Given the disabling intensity of symptoms and the radiological uncertainty, early surgical re-exploration was considered and ultimately performed. At that stage, chemical radiculitis was not strongly considered, as the clinical presentation closely mimicked mechanical recurrence. This reflects the difficulty of clinical decision-making in such situations.

In retrospect, a trial of conservative management, including epidural steroid injection, could have been considered, particularly in the setting of equivocal imaging findings. This underscores the importance of considering chemical radiculitis in the differential diagnosis to potentially avoid unnecessary surgical intervention. However, in patients presenting with severe and disabling symptoms associated with diagnostic uncertainty, clinical decision-making may still reasonably favor early surgical exploration.

This case is particularly illustrative, as it demonstrates how chemical radiculitis can closely mimic early recurrent disc herniation both clinically and radiologically, leading to potentially avoidable reoperation in real-world practice.

## Conclusions

Chemical radiculitis should be considered in cases of early postoperative radicular pain following lumbar discectomy, particularly in the absence of clear compressive pathology on imaging. This entity may mimic recurrent disc herniation both clinically and radiologically, potentially leading to unnecessary reoperation if not properly recognized. Careful correlation between clinical presentation and imaging findings is essential to guide appropriate management. In such cases, a conservative approach may lead to favorable outcomes and help avoid the risks associated with repeat surgery.

Increased awareness of this condition is crucial for spine surgeons to improve diagnostic accuracy and optimize patient care. However, as this is a single case report, these findings should be interpreted with caution and cannot be generalized. This case contributes to the existing literature by emphasizing the diagnostic pitfalls of early postoperative radicular pain and the importance of integrating clinical, radiological, and intraoperative findings in decision-making.
